# Northern Ireland Cohort for the Longitudinal Study of Ageing (NICOLA): health assessment protocol, participant profile and patterns of participation

**DOI:** 10.1186/s12889-023-15355-x

**Published:** 2023-03-10

**Authors:** Charlotte E Neville, Ian S Young, Frank Kee, Ruth E Hogg, Angela Scott, Frances Burns, Jayne V Woodside, Bernadette McGuinness

**Affiliations:** Centre for Public Health, School of Medicine, Dentistry and Biomedical Sciences, Institute of Clinical Science, Queen’s University Belfast, Grosvenor Road, BT12 6BJ Belfast, United Kingdom

**Keywords:** Ageing, Health assessment, Cohort study, Biomarkers, NICOLA

## Abstract

**Background:**

The Northern Ireland Cohort for the Longitudinal Study of Ageing (NICOLA) is a prospective, longitudinal study of a representative cohort of older adults living in Northern Ireland, United Kingdom. Its aim is to explore the social, behavioural, economic and biological factors of ageing and how these factors change as people age. The study has been designed to maximize comparability with other international studies of ageing thereby facilitating cross-country comparisons. This paper provides an overview of the design and methodology of the health assessment which was carried out as part of Wave 1.

**Methods:**

Three thousand, six hundred and fifty five community dwelling adults, aged 50 years and over participated in the health assessment as part of Wave 1 of NICOLA. The health assessment included a battery of measurements across various domains that addressed key indicators of ageing namely: physical function, vision and hearing, cognitive function, and cardiovascular health. This manuscript describes the scientific rationale for the choice of assessments, provides an overview of the core objective measures carried out in the health assessment and describes the differences in characteristics of participants who took part in the health assessment compared to those who did not take part.

**Results:**

The manuscript highlights the importance of incorporating objective measures of health in population based studies as a means of complementing subjective measures and as a way to advance our understanding of the ageing process. The findings contextualize NICOLA as a data resource within Dementias Platform UK (DPUK), the Gateway to Global Ageing (G2G) and other existing networks of population based longitudinal studies of ageing.

**Conclusion:**

This manuscript can help inform design considerations for other population based studies of ageing and facilitate cross-country comparative analysis of key life-course factors affecting healthy ageing such as educational attainment, diet, the accumulation of chronic conditions (including Alzheimer’s disease, dementia and cardiovascular disease) as well as welfare and retirement policies.

## Introduction

### Background to NICOLA

The Northern Ireland Cohort for the Longitudinal Study of Ageing (NICOLA) [1] is a population-based, nationally representative ageing cohort study of adults aged 50 years and over and living in Northern Ireland [2, 3]. It is the first large-scale, in-depth, longitudinal study of ageing in Northern Ireland, and is one of a family of similar ageing studies across the globe aiming to gain a better understanding of factors affecting health and social outcomes in our rapidly increasing ageing populations. Sampling procedures and study design have been described fully elsewhere [3]. In brief, 8283 community dwelling adults, aged 50 years or older and living in private households across Northern Ireland were recruited from a randomised, stratified sample of Northern Ireland addresses obtained from the Business Service Organization General Practitioner Register and stratified by geographical location and postcode, thus ensuring a representative sample. Those who were institutionalized or who lacked capacity to provide informed consent were not eligible to participate. Wave 1 of the study had three components: a computer assisted personal interview (CAPI) conducted in the participant’s home, a pen and paper self-completion questionnaire (SCQ) including a dietary questionnaire and a health assessment. The Wave 1 CAPI interviews, conducted between December 2013 and July 2016, included questions on pensions, employment, living standards, health aspects including service needs and usage, as well as social contact and formal and informal care. The self-completion questionnaire included questions on relationship quality, loneliness, stressful and traumatic life events, worry and alcohol intake. The current paper focuses on the protocol used within the Wave 1 health assessment which took place between January 2014 and August 2018. Although spouses aged under 50 years were interviewed as part of the CAPI mainly to provide couple- or household-level data, they are not included in the current analysis. A follow-up (Wave 2) of the cohort took place between May 2017 and March 2022 and included a CAPI, SCQ and a COVID-19 questionnaire. A health assessment was not conducted in Wave 2. The results from Wave 2 will be presented in a separate manuscript.

### Scientific Rationale/Importance of NICOLA health assessment

International comparability was a key consideration in the design of NICOLA in order to ensure adoption of best practice and allow cross-national comparisons of results. As such, many of the methods employed in NICOLA are closely harmonized with those of other longitudinal studies of ageing, including The Irish Longitudinal Study of Ageing (TILDA) [4], the English Longitudinal Study of Ageing (ELSA) [5], the EPIC Norfolk Eye study [6], the UK Biobank Eye Study [7] and the US-based Health and Retirement Study (HRS) [8]. Further to this, the data and/or meta-data from the health assessment (along with the CAPI and SCQ data) is deposited in various data repositories including the Dementias Platform UK (DPUK), UK Data Service (UKDS), UK Longitudinal Linkage Collaboration (UK LLC) and Gateway to Global Ageing (G2G) which hold similar data from other cohort studies; this helps to maximise data sharing and foster research collaborations. Such data repositories help facilitate pooled analyses of core topics and allow comparability of the NICOLA cohort to other studies of ageing. More locally, NICOLA provides a strong, ongoing evidence base which will be used to inform local policy by helping researchers and policy makers understand better the social, health and demographic challenges of our ageing society.

The health assessment conducted as part of NICOLA was a fundamental component of the study, specifically designed to complement the self-reported data obtained in the CAPI, providing a range of objective measures of the health of the older population of Northern Ireland. The information typically obtained from objective measures of health and function can often be very different to information obtained from subjective self-reports. Integrating both objective and subjective measures therefore enables us to validate the self-reported data, identify previously undiagnosed illnesses such as hypertension or diabetes and act as useful indicators of the early signs of decline in health or physical function prior to symptomatic disease.

### Research direction on health assessment content

The content of the health assessment crossed a range of clinical domains, also drawing on the expertise of a wide range of research disciplines. Priority was given to health domains that were known to be of most relevance to the ageing process and which could reliably be measured in a population based study such as NICOLA. A unique methodological feature of the NICOLA health assessment was the detailed assessment of ophthalmic health.

### Objective of this overview

The purpose of this paper is to present an overview of the design and methodology of the NICOLA Wave 1 health assessment. The findings presented encapsulate the core objective measures of health and wellbeing of older adults who took part in the NICOLA Wave 1 health assessment. The information presented can inform design considerations for other population based studies of ageing and overall will add to the global body of evidence regarding harmonization of health measurements in older adults.

## Methods

### Design of health assessment protocol

All NICOLA participants who completed the baseline home CAPI as detailed previously [3] were sent a letter inviting them to attend a health assessment. Based on the relatively small geographical area of Northern Ireland (14,130 km^2^),the population distribution and accessible transport network, the Wellcome Trust-Wolfson Clinical Research Facility (CRF) located at the Belfast City Hospital was deemed a suitable location to perform the health assessments. The duration of the CRF-based health assessment was approximately two to three hours. All assessments were undertaken by research nurses and research assistants who received comprehensive training in the methodologies and provided clear step-by-step instructions to all participants. To encourage participation, travelling expenses to and from the CRF were provided to participants. A more condensed nurse-led home assessment lasting approximately 2 h was offered to respondents who were unable or unwilling to attend the CRF. Participants were phoned prior to the nurse attending their home.

### Health assessment methods

A robust battery of standardised assessments of cardiovascular function, respiratory function, physical function including hand grip strength, balance, walk speed, visual health, hearing and cognitive health were used, all of which are comparable to those used in other longitudinal studies internationally. Other standard clinical measures including blood pressure, height, weight, and hip and waist circumference were also collected. Non-fasting blood and urine samples were also obtained as part of the health assessment. If glucose or lipid results were outside the normal expected range, then both the participant and participant’s General Practitioner were informed in writing. The assessment methods and their rationale for inclusion in the health assessment are detailed below.

Table 1 provides an overview of the physical, cognitive health, mental health, dietary assessment measures and biological samples measures included in the health assessment and compares the measures to other comparative longitudinal studies of ageing. While many of these measures are described in detail, a comprehensive description of the protocols used is beyond the remit of this article. Further manuscripts detailing specific strands of research being conducted within NICOLA that are not included in this manuscript will be forthcoming including the results from the analysis of the Wave 1 dietary questionnaire.

**Table 1:** Measures used in the NICOLA health assessment compared to other similar longitudinal studies of ageing

Outcome Measure	Type of assessment	Measures	Comparative study
Physical Health	Anthropometric	Weight Height Waist and hip circumference	TILDA, ELSA
	Body composition	Bodystat (% body fat)	None
	Cardiovascular	Blood pressure Orthostatic blood pressure	TILDA, ELSA
	Respiratory	Spirometry	ELSA
	Mobility and strength	Step test Timed up and go Grip strength (dynamometry)	TILDA, ELSA
	Vision	Visual acuity Multi-modal retinal imaging	TILDA (visual acuity)
	Facial photograph	Physical attractiveness / signs of ageing	None
Cognitive Health	Cognitive function	MMSE MOCA Colour trails 2 Animal recall	TILDA
Dietary Intake*	Food frequency questionnaire	Dietary intake (EPIC-FFQ) Special diets Cooking and food shopping Food supplements / vitamins	ELSA (Wave 9 only, online FFQ (Oxford-WebQ))
Mental Health	Mental well-being Depression	Warwick-Edinburgh Mental Well-Being Scale (WEMWBS) Centre for Epidemiologic Studies Depression Scale (CES-D)	None ELSA
Biological Samples	Blood and urine sample (non-fasting)	Lipid profile Genomic biomarkers Dietary biomarkers Bone markers Inflammatory markers Other biomarkers	TILDA, ELSA

* Not detailed in this paper

### Physical health

#### Body composition: height, weight, BMI, waist, hip, body fat

Changes in body composition are a normal part of ageing and often occur simultaneously with declines in physical function. Anthropometric measurements were made to provide a quantitative measure of body composition, obesity and body fat distribution that is related to overall health status and can be tracked over time. Standing height and weight were measured using standard techniques, BMI was computed as weight/height^2^ (kg/m^2^).

Waist and hip measurements were recorded using a SECA measuring tape. The waist was measured midway between the iliac crest and the costal margin (lower rib) while the hip circumference was measured at the widest circumference over the buttocks and below the iliac crest. Measurements were repeated twice. Waist-to-hip ratio was calculated as a measure of body fat distribution which is an important indicator of risk of cardiovascular disease [9]. Percentage body fat was also measured using the Bodystat 1500 MDD body composition analyser. This measures the amount of lean and fat mass that makes up total body weight.

#### Physical function – step test, timed up and go, grip strength

Physical function is one of the most important indicators of health status in older adults and is closely related to quality of life. Ageing is associated with numerous anatomical and physiological changes which can adversely affect physical function, thus contributing to an increased risk of falls, fractures and disability. At a population level, impaired physical function is known to be associated with frailty [10], increased mortality [11] and greater utilisation of health services [12].

In NICOLA, objective measures of strength, mobility and balance were used to capture overall physical function as they are robust early indicators of decline in physical function. These biomarkers can help provide an indication of future risk of many health conditions and loss of independence. They are therefore useful indicators of healthy ageing as well as being a sign that early intervention is required.

The ‘step up’ test was used to measure dynamic standing balance, combining a measure of balance and lower-extremity motor control [13]. It was recorded as the number of times the participant fully stepped on and off a 7.5 cm block step in 15 s. Measurements were taken for each leg and the number of times the participant stepped up was counted and averaged across the right and left feet. The greatest number of steps completed corresponded to better dynamic standing balance.

The timed up-and-go (TUG) test is a test of mobility commonly used in clinical practice to measure mobility and risk of falling [14, 15]. Impaired mobility often precedes the onset of physical disability, falls, frailty and cognitive impairment. Slower test speeds have been shown to be related to increased risk of health conditions and mortality in older adults [16]. The test measures the time taken by the participant to stand up from a standard arm chair, walk three meters at their usual pace, turn, walk back to the chair and sit down again [15]. It is a robust test of functional mobility as it assesses proximal muscle strength, balance, executive function and gait speed. Typical values range from 8 to 11.5 s with a faster time indicating greater mobility. A time greater than 12–15 s is often used as an indicator of a high risk of falling [17] and greater than 10 s an indicator of frailty [18]. Participants were permitted to use their usual assistive device such as a cane or walking aid, and. were also permitted to stop and rest (but not sit down) during the test, if required. .

Grip strength affects everyday function, such as the ability to hold heavy objects, and declines with age. A higher grip strength is associated with a reduced risk of early mortality, cardiovascular disease and disability [19]. It is also a good indicator of biological ageing [20]. Hand-grip strength was assessed using a Baseline hydraulic hand-held dynamometer. This method has previously been shown to be a reliable and valid instrument for assessing muscle strength and function [21–23] and is an indicator of frailty in older adults [24]. The participant stood with their forearm flexed at 90 degrees and squeezed the handle of the dynamometer with maximum force. Measurements were repeated twice with each hand, alternating between the dominant and non-dominant hand. The data presented represents the average of two tests using the dominant hand.

#### Cardiovascular function – blood pressure

Blood pressure is a modifiable risk factor for adverse cardiovascular events such as coronary heart disease and stroke. Hypertension is recognised as one of the most preventable causes of premature morbidity and mortality. The prevalence of both diabetes and hypertension increases sharply with age but can only be dealt with properly at a population level if we know how many go undiagnosed with these conditions. Evidence suggests that many older adults are unaware that they have hypertension. In the UK, 1 in 3 adults suffer from hypertension(a reading of 140/90 mm Hg or higher; [25] rising to at least 1 in 2 in those aged 65 years and over [26]. In addition, as a person ages, the tendency for postural hypotension (BP drop on standing) increases. This can result in dizziness, light headedness and increases the risk of falls. Systolic (SBP) and diastolic blood pressure (DBP) was measured using the OMRON TM digital automatic blood pressure monitor (Model M10-IT). Blood pressure and heart rate was measured three times (one minute apart) on either arm. The one-minute gap between blood pressure measurements was based on the 2005 AHA position statement [27] which recommended at that time, that at least two blood pressure readings should be taken at intervals of at least one minute and an average calculated. Given the pragmatic approach used in the design of the health assessment, a one-minute gap was also deemed more logistically feasible, in order to keep each assessment as short as possible for the participant. Two of the measurements were taken with the participant seated, while the third was recorded immediately upon standing (postural blood pressure). Hypertension was defined as SBP ≥ 140 mmHg or DBP ≥ 90 mmHg or current blood pressure-lowering treatment [25].

#### Respiratory function

The respiratory system undergoes various anatomical, physiological and immunological changes with age. Ageing is associated with a progressive decline in respiratory function that accompanies changes in the structure of the chest wall due to loss of supporting tissue, increased air trapping and decreased respiratory muscle strength [28]. Respiratory function was measured using the CareFusion Microlab Spirometer with the participant seated. Measurements included forced expiratory volume in one second (FEV1, l), forced vital capacity (FVC, l) and forced expiratory flow (FEF) 25–75%. Measures of lung function (FEV1 and FVC) are associated with all-cause and cardiovascular mortality [29, 30]. Low FEV1 is also recognised as an independent predictor of non-cardiopulmonary comorbidities including diabetes, chronic kidney disease, osteoporosis and dementia [31–34]. For the purposes of this manuscript the highest FEV1 and FVC reading was used. A maximum of five attempts were undertaken to obtain three satisfactory readings. Analyses are only based on participants who obtained at least three satisfactory readings.

#### Vision – visual acuity

Significant losses in visual function are known to occur with normal ageing. With increasing age, the incidence of eye diseases such as cataract, age-related macular degeneration (AMD), glaucoma, and diabetic retinopathy increases significantly. Globally, cataracts, glaucoma and AMD are leading causes of adult-onset blindness [35]. Although age is a major risk factor for visual loss, other risk factors include smoking, genetic tendency, pigmentation, arterial hypertension, ultra violet light exposure and consumption of an unbalanced diet. Even in the presence of relatively good visual acuity, decreases in visual function with age are related to a decreased quality of life, mobility and independence in older adults [36]. A unique strength of NICOLA compared to our comparative studies is our ability to exploit research areas such as eye health where we have core research expertise. To maximise capacity in this area of research and capitalize on our in-house expertise, the health assessment included an in-depth ophthalmic assessment comprised of two sections:

i) the Optometric assessment which evaluated visual function using distance visual acuity, refractive status using auto refractor (Shin Nippon Accuref K-900) and intra ocular pressure using the Ocular Response Analyser. Distance visual acuity measurements were performed in each eye. Habitual visual acuity was measured using the Early Treatment Diabetic Retinopathy Study (ETDRS) classification chart [37] and was recorded as the number of letters correctly identified from either the 4 m chart or the 1 m chart with and without a pinhole occlude. Participants wore their own glasses or contact lenses during the measurement. The ETDRS classification system is considered to be the gold standard for the measurement of visual acuity in clinical research and practice [38].
ii) Multi-modal retinal imaging using the Canon CX-1 Color Fundus digital camera (Canon USA, Inc.), Optus P200Tx wide field retinal imaging camera (Optos plc, Dunfermline, UK) and spectral domain optical coherence tomography (Spectalis HRA + OCT) (Heidelberg Engineering, Heidelberg, Germany).

Prior to the ophthalmic imaging, tropicamide 1% eye drops were applied to the pupil of each eye (or the non-dominant eye if preferred by the participant or if the participant was driving within 4 h or neither eye) of the participant in order to enlarge the pupil and thus achieve good quality retinal images. 626 (17%) participants did not give consent to have eye drops administered. While reduced pupil size impacts the quality of the colour fundus photographs (CFP) the most [39], OCT is more robust to pupil size. Obtaining multiple imaging types was therefore a strength of NICOLA compared to most epidemiological studies which usually only capture CFP. Images were acquired using stereo colour fundus photography centred on the disc and macula, a single non-stereo unsteered pseudocolour ultrawide field image, Autofluorescence (AF), MultiColor (MC), Macular OCT scan centred on the fovea and a circle scan of the optic disc.

Standardized multi-modal retinal grading by the Network of Ophthalmic Reading Centres UK [40] was used to identify features of common eye conditions such as AMD, glaucoma, diabetic retinopathy, vitreous interface changes and macular holes [39]. Features of AMD such as drusen type, size and location, the presence of hyper pigmentation, presence of focal or geographic atrophy or signs of retinal neovascularization were identified [41]. Participants were then classified into AMD grades, based on the Beckman Clinical Classification System which provides a severity scale for AMD spanning from no AMD to the most severe clinical manifestations which are accompanied by vision loss. [42] The AMD grades are: (i) No ageing changes (no drusen present and no AMD pigmentary abnormalities); (ii) Normal ageing (small drusen ≤ 63 μm present and no AMD pigmentary abnormalities); (iii) Early AMD (medium drusen > 63 μm and ≤ 125 μm and no AMD pigmentary abnormalities); (iv) Intermediate AMD (large drusen > 125 μm and/or any AMD pigmentary abnormalities); (v) Advanced AMD (neovascular AMD and/or any geographic atrophy) [42]. Diabetic retinopathy and maculopathy were also identified and graded from all retinal imaging modalities. The English Classification system was used to categorise participants according to level of severity [43].

A subset of participants who were suspected of having glaucoma due to optic disc appearance or raised intraocular pressure were also invited to a follow up health assessment for further evaluation of glaucoma by a glaucoma expert. Tests performed at this visit included (i) visual field testing using Humphrey’s Matrix frequency doubling technology (FDT) perimetry (Carl Zeiss Meditec Inc., Dublin, CA, USA) in low illumination, (ii) Gonioscopy and (iii) pupil dilation and biomicroscopy including optic disc examination [44, 45].

#### Hearing

Hearing loss is highly prevalent in older populations and is the most common sensory impairment in older adults [46]. If left untreated, hearing loss can have a profound impact on overall quality of life and everyday life through its effect on the ability to communicate and remain independent [47]. Untreated hearing loss also has indirect health, psychosocial, and economic effects thus resulting in increased feelings of loneliness, emotional distress, social isolation and withdrawal from social situations [48–51]. Those experiencing hearing loss are also likely to have other age-related conditions and are at greater risk of falls and frailty [52], as well as higher rates of cognitive decline [53–56]. Although not successful in everyone, hearing aids can improve several aspects of life that have been compromised by hearing loss. However, despite the availability of hearing aids and major technical progress in the last decade, uptake of hearing aids is poor and only a relatively small proportion of adults with hearing impairment seek help for their hearing problems and use hearing aids. In NICOLA,hearing was not measured objectively, but rather by self-report which assessed participant’s hearing ability, their use of hearing aids and coping with hearing problems including the impact of hearing loss on following conversation or using a telephone. A validation study of the self-report methods was also carried out separately in a subsample of NICOLA participants (n = 120) to examine the association between self-reported measures of hearing loss and measured hearing loss using pure-tone audiometry, the gold standard method of hearing loss assessment [57]. Low but significant correlation, and fair agreement using weighted kappa was found between self-reported measures of hearing loss and measured hearing loss by pure-tone audiometry [58].

#### Facial photograph

It has previously been suggested that life experiences are reflected in your face. For example, some people look younger in a photograph than they actually are. Participants were informed in advance, via the participant information sheet, that a photograph would be taken of them sitting in a chair and that the purpose of the photo was to see how appearance changes as people get older. Two facial photos in portrait format (one face-on, one side profile) were taken of each participant using a Nikon Coolpix L610 digital camera, in order to enable comparisons with other indicators of ageing. The photo captured the participant’s face, hair and part of the neck. The participant was asked to not smile in the photo and to remove glasses and headwear. Make-up and other items such as jewellery or hearing aids were permitted. The camera lighting was set in order to capture facial texture.

### Cognitive health

Preventing dementia and cognitive decline is a global health priority. In 2010, it was estimated that there were 35.6 million people with dementia worldwide [59]. It has been predicted that this figure will approximately double every 20 years [59]. Cognitive function outcomes were determined using a cognitive battery comprising four standardized measures which assessed memory, planning, attention and reasoning. These measures included a combination of pen and paper based tests or verbal tests, with responses being recorded by the research nurse. All cognitive tests were conducted in a quiet room and in a fixed order.

#### MMSE

The Mini-Mental State Examination (MMSE) was used to assess global cognition [60]. It consists of 30 brief questions (verbal and pen/paper based) which are designed to measure a range of cognitive domains including attention and concentration, memory, language, visuo-constructional skills, calculations and orientation. The MMSE took approximately 5 min to administer. A score (out of 30) based on performance across the 11 components of the test (orientation to time, orientation to place, registration, attention and calculation, recall, naming, repetition, comprehension, reading, writing and drawing) was calculated for each participant. Total scores ranged between 0 and 30, with lower scores indicative of more severe cognitive impairment and scores of 25 or over indicating no cognitive impairment.

#### MOCA

The Montreal Cognitive Assessment (MoCA) [61] is typically used as a rapid screening instrument for mild cognitive impairment. It is more sensitive than the MMSE to mild cognitive impairment [62]. It assesses different cognitive domains including attention and concentration, executive functions, memory, language, visuoconstructional skills, conceptual thinking, calculation and orientation. The test which included a combination of verbal and pen/paper based tests took approximately 5–10 min to complete. A score (out of 30) based on performance was calculated for each participant with lower scores indicating greater cognitive impairment and scores of 26 or over indicating normal cognitive functioning.

#### Colour trails 2

The Colour Trails 2 test was used to measure executive function and visual scanning. Participants were instructed to draw a line as quick as possible between consecutively numbered circles, but alternating between pink and yellow colours [63]. The length of time taken to complete the test was recorded in minutes, seconds and centi-seconds. The number of near misses, prompts, colour sequence errors and number sequence errors made by the participant was also recorded.

#### Animal Recall

Animal recall is a measure of executive function (e.g. strategic search and set-shifting) and semantic memory. Participants were asked to verbally name as many animals as possible within 60 s [64]. One point was given for each animal named. The number of animals named was recorded by the research nurse. Different species, genders or generations of animals were counted separately (e.g. dog, spaniel, bull, calf) but redundancies were not (e.g. brown cow, white cow). One point was allocated for each animal named by the participant with the total number reflecting verbal fluency score.

### Mental health

#### Warwick Edinburgh Mental Well-being Scale

The Warwick-Edinburgh Mental Wellbeing Scale (WEMWBS) is a scale of 14 positively worded items such as “I’ve been feeling interested in other people” and “I’ve been feeling good about myself” and is used to assess the mental health of the general population. For each statement, participants were asked to rate on a Likert scale of 1 to 5 how often they had felt like that, with one being “none of the time” and five being “all of the time”. Scores on the WEMWBS ranged from 14 to 70, with higher scores indicating higher levels of wellbeing. Participants self-completed the questionnaire, using pen-paper, in private and then returned the completed questionnaire to the research nurse in a sealed envelope. The WEMWBS has been validated for use in the UK in those aged 16 years and above [65] and specifically for the general population in Northern Ireland [66].

#### Centre for Epidemiological Studies Depression Scale (CES-D)

Depressive symptoms are known to influence cognition and it is important to control for mood when analysing cognitive results. However, many studies of ageing have excluded patients with depression from cognitive trials and vice versa in depression trials. It is important to be able to track the stability of a person’s mood over time and how changes in mood relate to future health status. The CES-D consists of 20 items phrased as statements, each one assessing symptoms associated with depression [67]. Participants had to verbally respondon a scale of 0 to 3 how often they had experienced that symptom over the previous 7-day period ranging from 0 (“rarely or none of the time”) to 3 (“most or all of the time”). Four of the items are positive statements which are inversely scored. Responses to each item were summed to generate a total score ranging from 0 to 60 with a higher score indicating a higher degree of depressive symptoms [68]. In general, a score of ≥ 16 is indicative of moderate or potentially clinically relevant depressive symptoms while a score of 8–15 indicates mild or sub-threshold depressive symptoms [67, 68]. The CES-D scale has been shown to be reliable at measuring the number, types and duration of depressive symptoms [68]. However, it is important to note that while the CESD is widely used across large scale population based epidemiological studies, it only assesses symptoms over the previous 7-day period rather than over a longer period of time. It is also considered to be a psychometric screening tool for depression and not a diagnostic tool [68].

### Dietary intake

A healthy diet is an integral part of healthy ageing and plays a key role in chronic disease prevention and in reducing the risk of cognitive decline [69–71]. Indeed, the importance of eating a healthy balanced diet as we get older cannot be underestimated as it protects against illness, helps to speed recovery from illness and importantly, maximizes the chances of living longer and independently in good health [72, 73]. However, the ageing process results in many physiological, social and psychological changes that can affect nutritional intake and status thus increasing the risk of malnutrition [73–76]. NICOLA is unique in that it is one of the few longitudinal studies of ageing which includes a detailed dietary assessment [77].

Exploring the effects of diet on the ageing process is a core focus of NICOLA. Dietary intake was assessed using the validated 130-item food frequency questionnaire (FFQ) (EPIC-Norfolk) (CAMB/PQ/6/1205) [78]. Participants were asked to record the frequency of consumption (never or less than once a month, 1–3 times per month, once a week, 2–4 times per week, 5–6 times per week, once a day, 2–3 times per day, 4–5 times per day, 6 + times per day) of standard portions of foods over the previous 12 month period. Additional components of the FFQ included questions relating to special diets, supplement use, eating outside the home and the cooking, preparation and shopping for food.

Given the uncertainty over the utility of a FFQ to determine dietary intake in older people, a validation study of the NICOLA FFQ was also incorporated into the design of the dietary assessment [79]. In addition to completing a FFQ and providing a blood sample, a subsample of the NICOLA cohort (n = 44 men and n = 51 women) also completed two food diaries as a reference method (6 months apart) and provided additional blood, urine and saliva samples for measurement of nutritional biomarkers. Of these 95, 23 participants also took part in multiple 24 h recalls.

Findings from the in-depth dietary analysis of the FFQ including energy and nutrient intakes, dietary patterns, dietary supplement use and the dietary validation study are beyond the remit of this paper and will become available in due course. This work will allow us to address the lack of dietary validation studies in older people to date and will allow us to test numerous hypotheses around diet-disease and diet-function relationships in older people.

### Biological samples

Analysing biological samples enables us to objectively evaluate biomarkers that act as an indicator of a person’s health. Biomarkers can also provide an early indication of disease before symptoms arise, provide us with information on disease progression and/or suggest therapies. In NICOLA, non-fasting venous blood samples were obtained from consenting participants. These included blood serum, plasma (EDTA/clot activator), glucose (potassium oxalate/sodium fluoride) and RNA (PAXgene). A spot urine sample was also obtained from all participants. All biological samples were transported in temperature controlled containers to a central laboratory and processed within 4 h. Aliquoted samples were subsequently frozen at − 80 °C until analysis. A dedicated courier service was used for transporting samples collected at the home-based assessments. As described previously, detailed laboratory analysis was conducted on all of the samples which included multi-omic biomarkers, lipid profiling, dietary biomarkers, inflammatory biomarkers and hormones [3]. All laboratory assays were standardised against available international standards, and quality control samples were included in every run. Participants consented separately for the collection of blood, DNA, urine, retinal images, facial photograph and the administration of the eye drops including consent for analysis, storage and future contact. Data are currently available for 28 biochemical biomarkers from 3082 participants within the NICOLA cohort. Participants were also offered rapid testing and feedback from blood glucose and lipid levels. NICOLA has a strong focus on molecular biomarkers and complementary genetic, epigenetic and transcriptomic data is available for a subset of participants. There is also 551,830 directly genotyped and 18,148,478 imputed SNPs currently available for 2969 participants.

### Study management

NICOLA is managed under the ethics and governance approval processes of Queen’s University Belfast. Ethical approval for the health assessment was granted by the School of Medicine, Dentistry and Biomedical Sciences Ethics Committee, Queen’s University Belfast. Written consent was obtained from all participants prior to participation in the health assessment.

The NICOLA data and sample resource is governed by the NICOLA Steering Committee, Data Access Committee and Research Support Team. The Steering Committee provides oversight on all research carried out on study participants and on data and advises on the best ways of optimising scientific potential. This interdisciplinary team includes experts across various research areas including chronic illness, physical activity, the built environment, nutrition, eye health, cognitive health, mental health, frailty, social environment, and multi-omics. Approved researchers, members of academia, and others from the third sector, practitioner, government and policy communities who wish to access the anonymised dataset can do so by making an application using the designated Research Proposal Form available on the study website. Research proposals to access data from the NICOLA resource must be in accordance with the NICOLA Data Access Policy and follow a standardised review and approval process by the NICOLA Data Access Committee. The approval structure includes regular operation of the Data Access Committee which oversees data access, review proposals, and tracks published papers and public engagements. Each separate research project is assessed against data governance criteria and a determination is made as to whether the outcomes meet the remit of the NICOLA study governance objectives. The Research Support Team is responsible for maintaining the security of the data and ensuring confidential access and for managing and curating the research data generated from NICOLA. All outputs generated from the data are subject to a disclosure control assessment. The NICOLA research support team currently manage the integration of study data with linked routine records, integrate the research application process and provide secure data access to research users.

All data is collected, stored and disseminated in accordance with the QUB Research Management Policy as well as in line with UK General Data Protection Regulations (GDPR), Data Protection Act (2018), Human Tissue Authority Codes of Practice and in accordance with the NICOLA Data and Sample Access Policy https://www.qub.ac.uk/sites/NICOLA/InformationforResearchers/#requesting-access-to-nicola-data-or-biological-samples-910951-1. The Data and Sample Access Policy describes in detail the general processes and procedures involved in accessing the NICOLA data resource (defined as data already collected and the participants themselves for the purposes of new data collection) and NICOLA samples (biological, clinical, and multi-omic). Within NICOLA, we aim to encourage and facilitate data access with all ‘bona fide’ researchers and research organisations as defined by UK Research and Innovation (UKRI) (https://www.ukri.org/) and welcome proposals from researchers, either for collaborative projects or for other forms of data access to help advance research knowledge.

### Statistical analysis

Descriptive statistics were obtained for all selected baseline variables of interest. Continuous and categorical variables were summarized as mean (SD) and n (%) respectively. Data where distributions were positively skewed are presented as median (interquartile range). Chi-square tests were used to compare categorical data. The main statistical analysis offered in this paper is designed for descriptive purposes [80]. For all comparisons, study participants have been classified according to type of physical health assessment: clinic based, home based or none. Characteristics of respondents were compared across visit type using analysis of variance for continuous measures and chi-square tests for categorical variables. Binary logistic regression was used to compare participants who attended the health assessment versus non-attendees. Values for the logistic regression analysis are presented as Odds ratio (95% CI). For all analyses, p < 0.05 was considered statistically significant. Statistical analyses were performed with SPSS v24.0 for Windows (SPSS Inc, Chicago, IL).

## Results

### Wave 1 Health Assessment Response Rates

Response rates are presented in Table 2. Of the participants who completed the Wave 1 CAPI, 44% (n = 3655) also took part in the health assessment. The majority of participants attended the Clinical Facility for the health assessment (95%, n = 3462), with the remainder taking place in the participants’ home (5%, n = 193). The majority (96%, n = 3514) of participants who attended the health assessment also consented to providing a venous blood and urine sample.

**Table 2:** Completion rates of the NICOLA Wave 1 health assessment

	Health Assessment		
Age group (yrs)	All (n)	Clinic Based (n)	Home Based (n)
50–64	1977	1937	40
65–74	1187	1125	62
≥ 75	491	400	91
Total	3655	3462	193

### Characteristics of health assessment attendees

Table 3 describes selected baseline demographic, anthropometric and biological characteristics of participants who attended the health assessment (either at home or at the clinic). The majority of participants who attended the health assessment were in the youngest age category (i.e. aged 50–64 years); had reached secondary level education; were married; retired; and were a non-smoker. Participants in the older age category (i.e. age ≥ 75 years), with a lower level of education, and single were less likely to take part in the health assessment. Almost all participants (99%) were of white ethnicity.

**Table 3:** Selected sociodemographic and physical characteristics of participants who completed the NICOLA health assessment, by gender

	All (n_max_=3655)	Men (n_max_=1761)	Women (n_max_=1894)	P value^1^
**Health Assessment location**				
Clinic Home	3462 (94.7) 193 (5.3)	1678 (95.3) 83 (4.7)	1784 (94.2) 110 (5.8)	0.14
**Age group (yrs)**				
50–64 65–74 ≥ 75	1977 (54.1) 1187 (32.5) 491 (13.4)	884 (50.2) 613 (34.8) 264 (15.0)	1093 (57.7) 574 (30.3) 227 (12.0)	< 0.001
**Marital status**				
Married / cohabiting Never married Separated / divorced / widowed	2643 (72.3) 244 (6.7) 768 (21.0)	1372 (77.9) 103 (5.8) 286 (16.2)	1271 (67.2) 141 (7.4) 482 (25.4)	< 0.001
**Employment**				
Retired Employed Self-employed (incl farming) Unemployed Permanently sick / disabled Looking after home / family / other^2^	1857 (50.9) 1059 (29.0) 369 (10.1) 86 (2.4) 141 (3.9) 137 (3.7)	908 (51.7) 439 (25.0) 254 (14.5) 59 (3.4) 73 (4.1) 23 (1.3)	949 (50.1) 620 (32.8) 115 (6.1) 27 (1.4) 68 (3.6) 114 (6.0)	< 0.001
**Education**				
Primary / none Secondary Higher	577 (15.8) 1602 (43.8) 1476 (40.4)	353 (20.0) 738 (41.9) 670 (38.0)	224 (11.8) 864 (45.6) 806 (42.6)	< 0.001
**Region**				
Urban Intermediate Rural	635 (17.4) 2048 (56.2) 964 (26.4)	283 (16.1) 1013 (57.7) 461 (26.2)	352 (18.6) 1035 (54.8) 503 (26.6)	0.09
**Multiple Deprivation Measure** ^**3**^				
1 **(**least deprived) 2 3 4 5 (most deprived)	991 (27.1) 763 (20.9) 733 (20.1) 658 (18.0) 510 (14.0)	498 (28.3) 360 (20.4) 353 (20.0) 296 (16.8) 254 (14.4)	493 (26.0) 403 (21.3) 380 (20.1) 362 (19.1) 256 (13.5)	0.26
**Anthropometric and body composition**				
Height (cm) Weight (kg) Body mass index (kg/m^2^) Body fat (%) Waist circumference (cm) Hip circumference (cm) Waist:hip ratio	165.6 (9.3) 79.7 (16.6) 29.0 (5.2) 44.2 (6.5) 95.8 (14.1) 104.7 (9.8) 0.91 (0.1)	172.6 (6.7) 87.2 (14.8) 29.2 (4.5) 44.7 (5.7) 101.9 (11.9) 104.5 (7.6) 0.97 (0.1)	159.1 (6.1) 72.7 (15.2) 28.7 (5.8) 43.8 (7.2) 90.2 (13.7) 105.0 (11.4) 0.86 (0.1)	< 0.001 < 0.001 0.002 < 0.001 < 0.001 0.06 < 0.001
**Blood pressure**				
SBP (mmHg) DBP (mmHg) SBP, postural (mmHg) DBP, postural (mmHg)	132.7 (18.8) 81.3 (10.9) 131.6 (20.4) 83.8 (11.2)	136.6 (17.8) 82.4 (10.9) 136.3 (20.1) 85.1 (11.1)	129.1 (19.1) 80.2 (10.9) 127.2 (19.7) 82.5 (11.1)	< 0.001 < 0.001 < 0.001 < 0.001
**Lipid and diabetes profile**				
Total cholesterol (mmol/l)	5.1 (1.2)	4.8 (1.1)	5.5 (1.1)	< 0.001
HDL cholesterol (mmol/l) Triglycerides (mmol/l) HbA1c (%)	1.5 (0.4) 1.5 (1.1, 2.1) 5.8 (0.9)	1.3 (0.3) 1.6 (1.1, 2.2) 5.8 (1.0)	1.6 (0.4) 1.4 (1.0, 2.0) 5.8 (0.8)	< 0.001 < 0.001 0.07
**Respiratory function**:				
Forced vital capacity (FVC) (l) Forced expiratory flow (FEV1) (l)	3.5 (0.9) 2.6 (0.7)	4.1 (0.9) 3.0 (0.7)	3.0 (0.6) 2.2 (0.5)	< 0.001 < 0.001
**Cognition**:				
MMSE (score) MOCA (score) Animal recall (number) Colour trails 2 (time in secs)	28.4 (1.8) 25.3 (3.3) 19.0 (5.6) 118.5 (41.5)	28.2 (1.8) 25.0 (3.2) 19.2 (5.7) 124.4 (45.2)	28.6 (1.8) 25.6 (3.3) 18.9 (5.5) 113.4 (37.4)	< 0.001 < 0.001 0.11 < 0.001
**Mobility and strength**:				
Step test Timed Up and Go (seconds) Grip strength (kg)	16.4 (4.3) 10.1 (2.8) 31.2 (11.8)	16.7 (4.3) 10.0 (2.6) 39.8 (9.8)	16.2 (4.2) 10.1 (3.0) 23.0 (6.4)	0.001 0.27 < 0.001
**Hearing**				
Use of hearing aid (self-reported)				
All of the time Some of the time	176 (4.8) 171 (4.7)	91 (5.2) 104 (5.9)	85 (4.5) 67 (3.5)	0.004
Quality of hearing (self-reported)				
Excellent / very good / good Fair / poor	2765 (76.0) 875 (24.0)	1222 (69.7) 530 (30.3)	1543 (81.7) 345 (18.3)	< 0.001
**Mental Health**				
CES-D (score) WEMWBS (score)	5 (2, 11) 55 (49, 61)	4 (2, 10) 55 (49, 61)	6 (2, 12) 55 (48, 61)	< 0.001 0.27
**Ophthalmic**				
Wear glasses or contact lenses Visited optometrist in last 12 mo Cataract (self-reported) Glaucoma (self-reported) AMD (self-reported) Visual acuity, distance, pin hole, better eye (number of letters) Refractive error, most severe eye (spherical equivalent from auto refractor)	3488 (95.8) 2242 (61.5) 775 (21.3) 92 (2.5) 90 (2.5) 82 (6) 0.83 (2.9)	1672 (95.2) 1026 (58.4) 344 (19.6) 45 (2.6) 51 (2.9) 83 (6) 0.93 (2.69)	1816 (96.4) 1216 (64.5) 431 (22.9) 47 (2.5) 39 (2.1) 82 (6) 0.74 (3.09)	0.11 < 0.001 0.01 0.89 0.11 < 0.001 0.06
Age-related macular degeneration^4^				
No AMD Normal ageing Early AMD Intermediate AMD Advanced AMD	1637 (51.4) 761 (23.9) 525 (16.5) 235 (7.4) 26 (0.8)	789 (51.3) 374 (24.3) 232 (15.0) 129 (8.4) 15 (1.0)	848 (51.6) 387 (23.5) 293 (17.8) 106 (6.4) 11 (0.7)	0.07
Glaucoma^5^				
Absent (both eyes)	3202 (96.9)	1549 (96.8)	1653 (97.0)	0.75
Present (either eye)	103 (3.1)	52 (3.2)	51 (3.0)	

Values are unweighted mean (SD) or median (IQR) for continuous variables or n (%) for categorical variables

^1^ p value represents difference between men and women

^2^ Other includes those in education/training (categories were combined due to low cell counts)

^3^ Based on the Northern Ireland Multiple Deprivation Measure 2010 [81]

^4^ Measurement based on retinal image and Beckman Clinical Classification

^5^ The International Society of Geographical and Epidemiological Ophthalmology (ISGEO) Classification

As presented in Table 3, significant differences were evident between men and women who attended the health assessment. Women tended to be in the youngest age group category (50–64 years); were more likely to be separated/divorced/widowed; to be employed or looking after home/family; and tended to have a higher level of education compared to men.

In terms of physical characteristics, blood pressure and lung function measurements (FVC and FEV1) were significantly higher in men who attended the health assessment than in women. Lung function measurements are typically higher in men than women although as well as sex, lung function also depends on age and height [82]. This will be examined in more detail at a later stage. Anthropometric measurements which included height, weight, BMI, body fat, waist circumference and waist:hip ratio were also higher in men than women. On average, NICOLA participants were overweight, with a mean BMI of 29.2 kg/m^2^ in males and 28.7 kg/m^2^ in females. Waist circumference measurements were also high in both men and women (101.9 cm and 90.2 cm, respectively). A waist circumference of ≥ 94 cm in men and ≥ 80 cm in women is associated with increased risk of developing obesity-related health problems. As well as measuring waist circumference, the ratio of waist to hip circumference is also used to indicate health risk. Mean waist:hip ratio was higher than the recommended level for both men and women (0.97 and 0.86, respectively). A waist-hip ratio > 0.90 and 0.85 for men and women respectively is associated with increased risk of a number of diseases including heart disease and Type 2 diabetes and is a better predictor of early mortality than BMI or waist circumference in older adults [9]. Grip strength was also higher in male attendees than women (39.8 kg and 23.0 kg, respectively). When examined by age category, age related decline in grip strength was greater for men than women (mean grip strength at age 50–64 years: men 43.8 kg, women 24.7 kg; age 65–74 years: 37.7 kg and 21.4 kg, respectively; age 75 + years: 31.2 kg and 18.3 kg, respectively) (data not shown). These values are within the expected normative values. For example, grip strength for a 65–75 year old is between 42.3 kg and 35.6 kg for men and between 25.3 kg and 21.4 kg for women [83]. This is consistent with the findings of TILDA [4, 84]. In terms of hearing and vision, a higher proportion of men (11%) reported using a hearing aid compared to women (8%) and reported that their hearing was fair/poor compared to women (30% and 18%, respectively). Likewise, TILDA similarly reported that men are more likely than women to use a hearing aid and to also report their hearing as fair/poor [84]. Visits to the optometrist in the previous 12 month period were higher in women than men (65% and 58%, respectively) and a higher proportion of women reported that they had been diagnosed with cataracts than men (23% and 20%, respectively). Just under 3% of both men and women reported a previous diagnosis of glaucoma and AMD. Based on the retinal image measurements rather than self-reported history, approximately three quarters of participants showed retinal changes consistent with normal ageing or no AMD (Class 0 or 1) while 16% had early AMD, 7.4% had intermediate AMD and 0.8% had advanced AMD in their worst eye. This is higher than the prevalence of AMD reported in the TILDA study [84] which had an estimated overall prevalence of 7.2%, with early / intermediate AMD accounting for 6.6% and late AMD accounting for 0.6%. Of the participants in the current study who were found to have advanced AMD, based on retinal imaging, approximately 40% did not report a positive history of having AMD (data not shown). Glaucoma was prevalent in approximately 3% of participants. These findings are comparable to pooled estimations of glaucoma prevalence in other European populations (based on age range 40–80 years) (2.93%, 95%CI 1.85, 4.40) [85]. Only 30% of participants who were found to have glaucoma during the health assessment had reported a positive history (data not shown).

In terms of cognition, the mean MMSE and MOCA score was 28.4 and 25.3, respectively. Similar to the findings from TILDA [83], the majority of participants (approximately 95%) showed normal levels of cognition (i.e. MMSE score 25–30) (data not shown). Performance in the colour trails 2 test showed the greatest difference between males and females, with females completing the test significantly faster than males (113 and 124 s, respectively). Similar to TILDA, symptoms of depression as reflected by the CES-D score were higher in women than men [84].

In terms of lipid profile, females had higher mean total cholesterol (5.5 mmol/l), HDL cholesterol (1.6 mmol/l) and lower triglycerides (1.4 mmol/l) compared to males (4.8 mmol/l, 1.3 mmol/l and 1.6 mmol/l, respectively). Mean cholesterol levels in females were higher than current recommendations which suggest that total cholesterol levels should be < 5 mmol/l in both men and women. HDL cholesterol levels should be ≥ 1.1 mmol/l in men and ≥ 1.2 mmol/l in women while non-fasting triglyceride should ideally be < 2.3 mmol/l [86].

Table 4 presents the difference in selected characteristics among those who had a home based health assessment, clinic based health assessment or no health assessment. When comparing characteristics across categories, those who attended the clinic for the health assessment tended to be 50–64 years old, women, married or cohabiting, living with others, retired, had secondary level of education, lived in an intermediate area in terms of urban/rural divide, were least deprived, were more likely to be a non-smoker, and a current consumer of alcohol. A greater proportion (44%) of those who attended the clinic based health assessment reported excellent or very good levels of health compared to those who opted for a home based assessment (23%) or who did not have a health assessment (32%).

**Table 4:** Selected characteristics of attendees (clinic-based *versus* home-based assessment) and non-attendees of the health assessment

	Odds ratio for attending health assessment (home or clinic)^1^	Clinic based health assessment (n = 3462)	Home based health assessment (n = 193)	No health assessment (n = 4628)	P value^2^
	Exp (B) (95% CI)	n (%)	n (%)	n (%)	
**Age group (yrs)**					
50–64 65–74 ≥ 75	Ref*** 0.97 (0.85, 1.11) 0.53 (0.45, 0.63)	1937 (56.0) 1125 (32.5) 400 (11.6)	40 (20.7) 62 (32.1) 91 (47.2)	2208 (47.8) 1309 (28.3) 1111 (24.0)	< 0.001
**Gender**					
Male Women	Ref*** 0.80 (0.73, 0.88)	1678 (48.5) 1784 (51.5)	83 (43.0) 110 (57.0)	1990 (43.0) 2638 (57.0)	< 0.001
**Marital status**					
Married / cohabiting Never married Separated / divorced / widowed	Ref 1.72 (0.70, 4.21) 1.93 (0.80, 4.65)	2557 (73.9) 227 (6.6) 678 (19.5)	86 (44.6) 17 (8.8) 90 (46.6)	2772 (59.9) 420 (9.1) 1436 (31.0)	< 0.001
**Living status**					
Living alone Living with others	Ref 2.27 (0.94, 5.45)	908 (26.2) 2554 (73.8)	107 (55.4) 86 (44.6)	1876 (40.5) 2752 (59.5)	< 0.001
**Employment** ^**3**^					
Retired Employed/Self-employed Permanently sick/ disabled Looking after home / family In education / training / other	Ref*** 0.83 (0.72, 0.95) 0.74 (0.54, 1.01) 0.44 (0.34, 0.53) 0.50 (0.38, 0.64)	1712 (49.6) 1413 (40.9) 122 (3.5) 174 (5.0) 35 (1.0)	145 (75.1) 15 (7.8) 19 (9.8) 14 (7.3) 0 (0)	2327 (51.2) 1303 (28.6) 463 (10.2) 420 (9.2) 35 (0.8)	< 0.001
**Education**					
Primary / none Secondary Higher	Ref*** 1.71 (1.51, 1.94) 2.54 (2.21, 2.92)	511 (14.8) 1504 (43.5) 1444 (41.7)	63 (32.6) 98 (50.8) 32 (16.6)	1529 (33.6) 2034 (44.6) 993 (21.8)	< 0.001
**Region**					
Urban Intermediate Rural	Ref*** 0.78 (0.68, 0.89) 0.67 (0.58, 0.78)	612 (17.7) 1754 (50.7) 1096 (31.6)	26 (13.5) 99 (51.3) 68 (35.2)	733 (15.8) 2304 (49.8) 1591 (34.4)	0.04
**Multiple Deprivation Measure** ^**4**^					
1 (least deprived) 2 3 4 5 (most deprived)	Ref*** 0.98 (0.85, 1.14) 0.77 (0.66, 0.88) 0.82 (0.70, 0.94) 0.62 (0.53, 0.72)	962 (27.8) 733 (21.2) 696 (20.1) 600 (17.3) 471 (13.6)	29 (15.0) 30 (15.5) 37 (19.2) 58 (30.1) 39 (20.2)	825 (17.8) 756 (16.3) 1017 (22.0) 963 (20.8) 1067 (23.1)	< 0.001
**Self-reported health**					
Excellent Very good Good Fair Poor	Ref*** 0.94 (0.80, 1.10) 0.89 (0.76, 1.05) 0.76 (0.64, 0.90) 0.60 (0.48, 0.74)	522 (15.1) 997 (28.8) 1057 (30.6) 648 (18.7) 235 (6.8)	12 (6.2) 32 (16.6) 39 (20.2) 63 (32.6) 47 (24.4)	428 (9.4) 1020 (22.3) 1245 (27.3) 1153 (25.3) 719 (15.7)	< 0.001
**Smoking status**					
Current Former Never	Ref*** 2.01 (1.73, 2.34) 1.97 (1.70, 2.29)	349 (10.1) 1294 (37.4) 1816 (52.5)	32 (16.6) 76 (39.4) 85 (44.0)	980 (21.5) 1498 (33.0) 2066 (45.5)	<0.001
**Alcohol status**					
Current Former Never	Ref*** 0.74 (0.66, 0.85) 0.81 (0.71, 0.92)	2408 (69.6) 491 (14.2) 561 (16.2)	80 (41.4) 53 (27.5) 60 (31.1)	2455 (54.1) 1045 (23.0) 1040 (22.9)	< 0.001

***p < 0.001 for difference between socio-demographic and health characteristics and odds of health assessment attendance

^1^ Each odds ratio controls for the other variables included in the table

^2^ P value is the statistical difference between groups

^3^ Some categories were combined due to low cell counts

^4^ Based on the Northern Ireland Multiple Deprivation Measure [81]

In contrast to the clinic based assessment, those who opted for a home based health assessment tended to be older (aged 75 years or over), separated/divorced/widowed, living alone, were more socially deprived, and had fair or poor self-reported health. Compared to those who attended the clinic, a higher proportion who opted for the home assessment were women, retired and with secondary education. Thedifferences in characteristics of participants depending on the location of the health assessment are somewhat consistent with those observed by TILDA with the exception of smoking status. In TILDA, respondents who chose to have a home based assessment were more likely to be a current smoker [4]. Just over a third (35%) of those who opted for a home based health assessment were from a rural area versus 13.5% of those who resided in an urban area. In comparison, TILDA reported that 13% of rural participants opted for a home assessment compared to 7.8% of those from an urban area [4].

Those who did not undertake a health assessment were more likely to be 50–64 years old, women, married/cohabiting, living with others, retired, had secondary education, living in an “intermediate” area i.e. other city or town outside the city of Belfast, were more deprived, had good self-reported health, were a non-smoker and currently consumed alcohol.

Table 4 also shows the association between population characteristics and the odds of the participant taking part in the health assessment, regardless of whether it was conducted at home or at the clinic. The likelihood of attending the health assessment (either home or clinic) was significantly higher in the youngest age category (i.e. 50–64 years), in males, retired, in those with a higher level of education and who rated their health as excellent. Participation rates in the health assessment were higher in those who were less deprived and lived in an urban area. Respondents were also twice as likely to be a former smoker (or non-smoker) and more likely to consume alcohol.

Table 5 shows the differences in selected physical and biological characteristics of participants who opted for a home based health assessment compared to a clinic based health assessment. Those who opted for a home based health assessment were shorter in height, had a higher percentage body fat, waist and hip circumference and waist:hip ratio compared to those who attended the clinic for the health assessment. Similar to TILDA, BMI and SBP was also higher in those who opted for a home based health assessment. Lipid profiles differed between home-assessed participants compared to clinic-assessed with lower levels of total cholesterol and HDL cholesterol and higher levels of HbA1c in those who had a home based health assessment. TILDA also reported lower levels of total cholesterol in home-assessed participants [4]. Similar to TILDA [4], levels of cognition, psychological health, and physical function were also lower in those who had a home based health assessment.

**Table 5:** Differences in selected physical characteristics of participants according to type of health assessment i.e. clinic-based *versus* home-based

	Clinic based health assessment (n_max_=3462)	Home based health assessment (n_max_=186)	P value^1^
**Anthropometric and body composition**			
Height (cm) Weight (kg) Body mass index (kg/m^2^) Body fat (%) Waist circumference (cm) Hip circumference (cm) Waist:hip ratio	165.8 (9.2) 79.7 (16.6) 28.9 (5.1) 44.0 (6.4) 95.6 (14.0) 104.6 (9.6)) 0.91 (0.09)	161.8 (9.3) 78.8 (18.1) 30.1 (6.6) 47.9 (7.8) 100.0 (15.6) 107.1 (13.0) 0.93 (0.09)	< 0.001 0.46 < 0.01 < 0.001 < 0.001 0.02 < 0.01
**Blood pressure**			
SBP (mmHg) DBP (mmHg) SBP, postural (mmHg) DBP, postural (mmHg)	132.5 (18.7) 81.4 (10.9) 131.4 (20.2) 80.4 (12.4)	137.2 (20.9) 78.1 (11.8) 134.6 (23.0) 84.0 (11.1)	< 0.01 < 0.001 0.08 < 0.001
**Lipid and diabetes profile**			
Total cholesterol (mmol/l) HDL cholesterol (mmol/l) Triglycerides (mmol/l) HbA1c (%)	5.1 (1.2) 1.5 (0.4) 1.5 (1.1, 2.1) 5.8 (0.9)	4.6 (1.3) 1.3 (0.4) 1.5 (1.1, 2.2) 6.1 (1.1)	< 0.001 < 0.001 0.05 < 0.001
**Cognition**:			
MMSE (score) MOCA (score) Animal recall (number) Colour trails 2 (time in secs)	28.5 (1.7) 25.5 (3.1) 19.2 (5.5) 116.5 (39.4)	26.8 (2.7) 22.2 (4.2) 15.3 (5.2) 165.0 (60.4)	< 0.001 < 0.001 < 0.001 < 0.001
**Mobility and strength**:			
Step test Timed Up and Go (seconds) Grip Strength (kg)	16.6 (4.2) 10.0 (2.7) 31.5 (11.8)	13.0 (4.1) 13.1 (4.8) 25.0 (10.6)	< 0.001 < 0.001 < 0.001
**Mental Health**			
CES-D (score) WEMWBS (score)	5 (2, 11) 55 (49, 61)	8 (3, 16) 52 (43, 59)	< 0.001 < 0.001

Values are unweighted mean (SD) or median (IQR).

^1^P value for statistical difference between groups

## Discussion

NICOLA is the first large scale longitudinal study of ageing in Northern Ireland, providing a basis for future government policy by following the trajectories of ageing in 8,500 men and women aged 50 years and over. The study adopts a conceptual framework [87], approach and methods that are closely aligned to other large scale longitudinal studies of ageing across the world including the HRS, ELSA, and TILDA [4, 5, 8], thus allowing cross-national comparisons of the NICOLA findings with those from other studies. Enabling comparative studies and learning from best practice is important for identifying local population needs and informing the modernisation of health and socio-economic policies and public services for older adults [88, 89].

The work presented within this paper demonstrates the multi-disciplinary nature of NICOLA and describes the scientific rationale for the choice of health assessments as well as providing an overview of the design and methodologies used in conducting the health assessment component of the study. The information presented can inform other population based studies of ageing in relation to study design and incorporation of objective measures of health into methodologies. The scope and wealth of data obtained will help contribute to the global body of evidence regarding harmonization of health measurements in older adults.

This paper also highlights the marked differences in characteristics of participants who attended the clinicbased health assessment compared to those who had a home based health assessment or no health assessment. Attendance at the health assessment clearly depended on the demographic characteristics, health and wellbeing of the respondents. Indeed there were marked differences in characteristics of participants who opted for a home based health assessment compared to those participants who travelled to Belfast for the clinic based health assessment. Significant differences were also evident between those who took part in the health assessment (either at home or clinic) compared to those who declined to take part in the health assessment in terms of demographic characteristics, behavioural factors, physical function and health status.

Respondents who attended the health assessment (either at home or clinic) were more likely to be younger (i.e. 50–64 years), male, retired or self-employed, have a higher level of education, and rate their health as excellent. Participation rates in the health assessment were higher in those who lived in an urban area with low levels of deprivation. Health assessment participants were also twice as likely to be a non-smoker (or former) and more likely to consume alcohol. The differences observed are consistent with those reported in other longitudinal cohort studies. Indeed, in cohort studies of older adults, age and cognition have been identified as the two main contributing factors to non-participation [90].

The findings presented highlights the importance of offering participants a home based assessment or a clinic based assessment. Offering participants the option of a home based health assessment helps to boost participation rates and helps to avoid potential under representation of older and more frail participants, particularly those who have mobility problems. However, while including a home based option might help optimize participation in the health assessment it nonetheless has limitations in terms of the breadth and scope of measures that can be undertaken. Clinic based health assessments can help facilitate a much broader and detailed physical health assessment.

Those who chose not to participate in the health assessment also represent a distinct group of older adults. Despite the robust sampling strategy within NICOLA, the difference in characteristics of those who took part in the health assessment versus those who did not take part highlights a need to target future recruitment strategies at certain demographic groups in order to ensure better representation of the population. Weights have subsequently been derived within the dataset to allow for these systematic differences in response and to ensure that estimates derived from the sample in different analyses remain representative of the Northern Ireland older population. These weights are based on factors which were shown to affect the likelihood of attending the health assessment including: age, sex, education, marital status, self-reported health, smoking status, alcohol status, location (Belfast; city or town; rural) and income domain score.

### Strengths of the NICOLA data and bioresource

The data from the NICOLA health assessment will provide a more comprehensive picture and understanding of the health challenges faced by today’s older adults and provide a discovery platform for researchers to try to unravel and address these challenges. The combination of objective and subjective data can shed light on the underlying mechanisms and pathways to sustained health as we age, and tell us more about the relationships between our biology, our lifestyle and our health outcomes. The findings will also undoubtedly provide a key knowledge base for decision makers developing and prioritising policy initiatives that are core to the health and wellbeing of older populations.

The value of NICOLA lies in its longitudinal design and large sample size. Without this, it is impossible to understand the crucial drivers of trajectories of ageing in Northern Ireland. The longitudinal design of NICOLA makes it well placed to continuously monitor changes in the trajectory of ageing and health status of older adults and review the impact of health policies on outcomes in Northern Ireland. As we follow up the NICOLA participants into old age, the insights will be further enriched, therefore the full potential of the data resource has yet to be exploited. Further in-depth research on various health domains and identification of novel biomarkers of ageing is ongoing.

The generation of molecular biomarkers and availability of rich multi-omic data within NICOLA’s bioresource provides a powerful resource. The generation of genetic-epigenetic-transcriptomic data, linked to biochemical biomarkers and extensive phenotype information, will help facilitate a broad spectrum of research. To date, NICOLA’s bioresource has helped identify multiple biological markers associated with more than 30 different phenotypes [91]. NICOLA has also contributed to developing innovative new approaches for multi-omic analyses, critically highlighting the importance of careful DNA and RNA storage for robust experimental studies. Early detection of declining health, particularly in the asymptomatic stages, is very important to facilitate early interventions that promote health and minimise loss of function and NICOLA is already identifying novel biomarkers for cardiovascular, eye, and kidney-related outcomes [91–95]. The combination of psychosocial phenotypes derived from our CAPI and the bioresource is also facilitating exploration of how social experiences and life adversity, for example social disadvantage, stressful exposures or traumatic events which is captured within the CAPI impacts the epigenome and health outcomes. This work is exploring how life circumstances in both childhood and adulthood affect epigenetic change and how different historical and life-course events and experiences influence health outcomes and the rate at which we age. Biological markers identified through this work could then be used to promote and maximise healthy ageing. Through this work we are also examining whether epigenetic changes are a cause or a consequence of particular ageing trajectories [95]. This research provides the opportunity for NICOLA to harmonise data with other international cohort studies and generate new molecular data.

### Future work

The NICOLA study will continue to expand its global impact and breadth of research. One such example is its current involvement in a new global collaboration to support cross-national research into dementia as part of a US National Institute of Health (NIH) grant for Harmonizing Cognitive Assessments in Irish, English and American Longitudinal Studies. An additional remit of this research involves exploring mechanistic pathways of cognitive health in relation to the built environment. This is being conducted as part of an ESRC funded Social Behavioural Design Research programme entitled Supportive environments for Physical and social Activity, healthy ageing and CognitivE health (SPACE). Overall, this work will help expand research into the epidemiology of cognitive decline and dementia and will contribute to global harmonisation of cognitive data thus providing new approaches towards prevention and potential treatment of Alzheimer’s disease and related dementias.

In-depth research on various other health domains is ongoing and the identification of biomarkers of ageing continues to be a major avenue of ongoing work with growing partnerships and joint funding. Data linkage and data harmonisation is also a focus of current work (details will follow in a separate manuscript). Further reports from the health assessment, in particular, more in-depth findings from the analysis of the retinal images [39], FFQ [784] and dietary validation study [73], will be forthcoming as well as bespoke reports on other specific age-related topics. Anonymised data from Wave 1 (CAPI, SCQ and Health Assessment), Wave 2 (CAPI, SCQ and COVID questionnaire) are now available for researchers to access. Further information regarding the application process for accessing data (Waves 1 and 2) and/or biological samples (Wave 1) is available on the researcher section of the NICOLA website [1].

Wave 3 of the study will commence later in 2023 which will continue the trajectory of longitudinal data collection and development of this data resource. This third wave will involve a follow-up of the current cohort of NICOLA participants (i.e. those who participated in Wave 2) and will involve a repeat health assessment, CAPI and SCQ with a focus on COVID immune response, microbiome, digital inclusion, food insecurity and eye health. However, for Wave 3, it is our intention to conduct a home based health assessment rather than participants attending the hospital facility. Due to the increasing age of the cohort, home visits have been deemed more acceptable and feasible and will help to reduce the burden on the participant. This awareness has come through informal feedback from current NICOLA participants with many indicating a preference for a home visit. Alternatively, participants will still be given the option of attending a clinicsetting in Wave 3 if they do not wish to have a home visit. Based on Waves 1 and 2, we have also identified a specific need to collect more bespoke data and samples as part of the Wave 3 health assessment including the analysis of the microbiome and COVID antibodies to further enhance the value of the study. The continued focus on COVID into Wave 3 will allow us to uniquely contribute to the path to post COVID recovery and to the rich and developing suite of Longitudinal Population Studies across the UK.

## Conclusion

In summary, this manuscript documents the scientific and methodological processes involved in the development and conduct of the health assessment component of NICOLA Wave 1 and highlights the difference in characteristics of participants taking part. The objective measures of the NICOLA health assessment allow innovative exploration of ageing including greater understanding of the ageing process and its determinants. Data from future waves of NICOLA will further enrich this data resource and will provide information relating to trajectories of health related to ageing.

## Data Availability

Researchers can apply for access to the data and biosamples by submitting a Research proposal to the NICOLA Data Access Committee. For more information, please refer to https://www.qub.ac.uk/sites/NICOLA/InformationforResearchers/.

## Abbreviations

AMD: age-related macular degeneration
CAPI: computer assisted personal interview
CES-D: Center for Epidemiological Studies Depression
CFP: colour fundus photography
CRF: Clinical Research Facility
DBP: diastolic blood pressure
DPUK: Dementias Platform UK
ELSA: English Longitudinal Study of Ageing
ETDRS: Early Treatment Diabetic Retinopathy Study
FDT: frequency doubling technology
FEF: forced expiratory flow
FEV1: forced expiratory volume in 1 s
FFQ: food frequency questionnaire
FVC: forced vital capacity
G2G: Gateway to Global Ageing
HDL-cholesterol: high density lipoprotein cholesterol
HRS: Health and Retirement Study
MMSE: Mini-mental state examination
MOCA: Montreal cognitive assessment
NICOLA: Northern Ireland Cohort for the Longitudinal Study of Ageing
OCT: optical coherence tomography
SBP: systolic blood pressure
SCQ: self-completion questionnaire
TILDA: The Irish Longitudinal Study of Ageing
TUG: timed up and go
UKDS: UK Data Service
UK LLC: UK Longitudinal Linkage Collaboration
WEMWBS: Warwick Edinburgh Mental Wellbeing Score
